# Oral hyperpigmentation as an adverse effect of highly active antiretroviral therapy in HIV patients: A systematic review and pooled prevalence

**DOI:** 10.4317/jced.60195

**Published:** 2023-07-01

**Authors:** Desiana Radithia, Ajiravudh Subarnbhesaj, Nurina-Febriyanti Ayuningtyas, Reiska-Kumala Bakti, Fatma-Yasmin Mahdani, Aulya-Setyo Pratiwi, Naqiya Ayunnisa, Salsabila-Fitriana Putri, Selviana-Rizky Pramitha

**Affiliations:** 1 Department of Oral Medicine, Faculty of Dental Medicine, Universitas Airlangga, Surabaya 60132, Indonesia; 2 Department of Oral Biomedical Science, Division of Oral Diagnosis, Faculty of Dentistry, Khon Kaen University, 40002, Thailand; 3 Bachelor Dental Science Program, Faculty of Dental Medicine, Universitas Airlangga, Surabaya 60132, Indonesia; 4 Oral Medicine Specialist Study Program, Faculty of Dental Medicine, Universitas Airlangga, Surabaya 60132, Indonesia

## Abstract

**Background:**

Human Immunodeficiency Virus (HIV) infects patients via CD4+ cells which are later be destroyed subsequently causing the deteriotation of immune system. HIV generally manifests in the oral cavity as the first indicating sign and a marker of disease progression. HAART medications are used to reduce the incidence of oral manifestations, however it can also generate adverse effects in the oral cavity including oral hyperpigmentation. This review aimed to estimate the prevalence of oral hyperpigmentation which affect individual quality of life as a side effect of HAART.

**Material and Methods:**

This systematic review applied Preferred Reporting Items for Systematic Review and Meta-Analyses (PRISMA) 2020. Literature search was performed in ScienceDirect, PubMed, and Scopus by combining terms such as highly active antiretroviral therapy, oral manifestation, epidemiology or prevalence published between January 1998 to March 2022.

**Results:**

Of 108 articles, eleven articles were included for systematic review and meta-analysis. The pooled prevalence of oral hyperpigmentation in HAART patients was 25% (95% CI: 11%, 38%; I2: 99%). Subgroup analysis based on geographical location showed varied result may be due to the type and duration of HAART used in study population. The most widely used type of ARV was from the NRTI group (n=7) and the study with the shortest duration showed the lowest oral hyperpigmentation prevalence (n=7).

**Conclusions:**

There is an increased prevalence of oral hyperpigmentation by the use of HAART. Future study should investigate the correlation between HAART duration and the degree of oral hyperpigmentation.

** Key words:**HAART, oral hyperpigmentation; pooled prevalence.

## Introduction

Human Immunodeficiency Virus (HIV) is a type of virus that may deteriorate individual immune system as the result of CD4+ cells destruction after being infected (1). HIV/AIDS itself has been classified as an epidemic with a total population of people living with HIV is approximately 34.0 million (31.4 million–35.9 million) according to the global epidemiological report on AIDS by the United Nations (UN) (2).

In HIV infected patients, decreased immune system commonly manifests in the oral cavity as the first indicating symptom and also a marker of disease progression (3). The most common oral manifestations that occur in HIV individual are candidiasis, hyperpigmentation, angular cheilitis, gingivitis, periodontitis, aphthous ulcers, herpes simplex infection, and oral hairy leukoplakia (2). HIV can infect people through several phases in which the final phase progresses to AIDS when appropriate management is not delivered immediately. The AIDS phase occurs due to the increased of viral replications so that the patient’s immunity decreases. In addition, patients with AIDS can also be infected with opportunistic diseases and presented with other clinical symptoms (4). Oral manifestations that occur can trigger more viral replication so that it can worsen the condition of HIV itself (5).

Most HIV patients are currently treated with highly active antiretroviral therapy (HAART) drugs. According to Nittayananta *et al*. (2010), it is known that there are good results on the frequency and severity of opportunistic diseases in HIV patients after using ART drugs (6). So far, there has been no research that proves that HIV patients can recover completely, but antiretroviral therapy may control the virus replication in the body (7). In mid-1995, antiretroviral drugs were developed in a treatment regimen consisting of three or more antiretroviral drugs which is then referred to as HAART (8). Based on Ponnam *et al*.(2012) and Rao *et al*.(2015) studies, HAART medications were found to be able to reduce the number of oral manifestations, particularly oral candidiasis and oral hairy leukoplakia (2,9). Despite the advantage of HAART in reducing many HIV oral manifestations, long-term use of HAART can also trigger an increase in oral hyperpigmentation (10,11).

Oral hyperpigmentation is clinically described as localized or generalized zones of blue to ill- defined black pigmentation affecting any site in the oral cavity, most commonly the gingiva, tongue, and buccal mucosa (12). This condition was found to be more predominant in HIV-patients on HAART than those who did not take HAART (3,6). Even though characterized as asymptomatic, oral hyperpigmentation could affect patients’ quality of life by causing aesthetic problems and has been associated with numerous systemic therapeutic agents. However, the specific pathogenesis of tissue pigmentation differs significantly and has yet to be discovered (11–13).

Considering the lack of discussion upon oral hyperpigmentations although it is common in HIV patients taking HAART, authors decided to conduct a systematic review and meta-analysis aiming to estimate the prevalence of oral hyperpigmentation as a side effect of HAART.

## Material and Methods

This systematic review followed the Preferred Reporting Items for Systematic Review and Meta- Analysis (PRISMA) 2020 (14).

-Eligibility criteria

Articles reporting oral hyperpigmentation in people with HIV positive on HAART at any age or gender were considered to be eligible. The included articles are those published in English from January 1998 to March 2022 using observational analytic design, such as longitudinal, cross-sectional, case controls, and cohort studies. Full text format should be able to be retrieved.

Article discussing oral pigmentation in HIV patients that appeared before or without HAART usage were not included. The combination of HAART with any other therapy, such as antitubercular, antifungal, antimalarial, antimicrobial, and chemotherapeutic agents were excluded. Study design considered to be ineligible were literature reviews, books, experimental studies, case reports, case series, conference papers, and guidelines.

-Search strategy

Literature search was performed by three reviewers (NA, SFP, SRP ) on March 20th 2022 in three databases, namely ScienceDirect, PubMed, and Scopus. Combinations of keywords and boolean operators (AND or OR) including Highly Active Antiretroviral Therapy (HAART, cART), oral hyperpigmentation OR oral hypermelanosis OR oral hypermelanoses OR oral melanosis OR oral melanoses OR oral melanism OR oral pigmentation OR melanotic macule OR HIV-associated melanin hyperpigmentation OR melanotic hyperpigmentation, AND epidemiology OR prevalence were applied to specify the search and determined which article to be included. Study design was filtered to observational analytic studies including cohort and cross-sectional studies.

**Figure 1:**
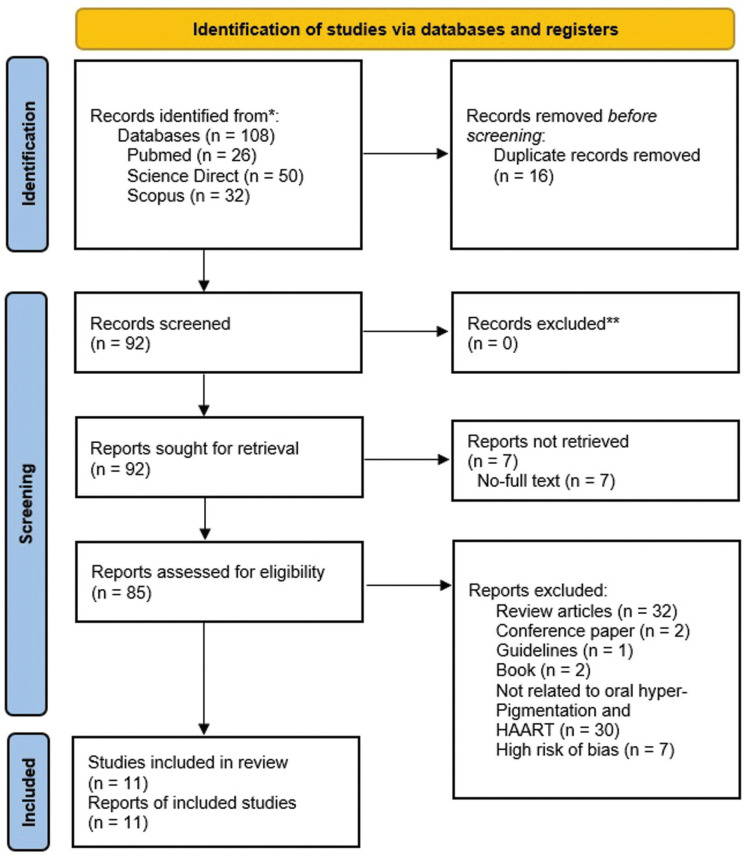
Preferred Reporting Items for Systematic Review and Meta-Analysis (PRISMA) Flow Diagram 2020.

The selection process adjusts to the predefined inclusion criteria. All data were double-checked by SRP for accuracy after the initial abstraction. Any disagreements were resolved by discussion with DR, NFA, and FYM as appropriate. The selection process is summarized following the PRISMA flow diagram (Fig. 1). After completing the article selection process, critical appraisal was conducted, (Fig. 1).

-Risk of bias assessment

Eligible articles were assessed for bias using an adapted Newcastle-Ottawa Scale that are relevant to cross-sectional studies. Studies with a score of 9-10 will be rated as very good studies and considered to have a low risk of bias. Studies scored 7-8 will be assessed as a good study. Studies with a score of 5-6 will be classified as satisfactory studies and a score of 0-4 will be assigned to studies that have a high risk of bias or studies that are unsatisfactory (15,16).

The assessment was carried out separately by two reviewers (NA, SFP) using Newcastle-Ottawa Scale. If there is any difference in the assessment between the two, the results of the assessment will be consulted on the third reviewer (SRP). The risk of bias assessment and critical appraisal are attached below in a Table form.

-Data extraction and synthesis

Data were extracted from included studies into spreadsheet by three reviewers (NA, SFP, SRP) and double-checked (SRP) to collect information such as author, study design, sample size, sample age, location and population, type of HAART, duration of HAART, and prevalence of OMP. The form also included a column for risk of bias assessment result. The standard error (SE) of prevalence was calculated from the reported percentage prevalence and sample size for each of the studies. SE was calculated as √ [p × (1−p)/n] (17). Prevalence of OMP was pooled using generic inverse variance random effect. Subgroup analyses were considered in reporting the continuous effect of oral melanosis by splitting population based on geographic location. Sensitivity analysis was performed by excluding study with high risk of bias (18).

## Results

-Search Results

The search results return a number of 11 articles using a cross sectional study design. The search was conducted using 3 formal databases, which were identified based on the criteria for side effects of ART on oral manifestations of HIV patients. The articles searched were only for the period 1998 - 2022. Of the 11 articles obtained, they had the full text that could be reviewed and included. The screening process is detailed in Table 1.

**Table 1:**
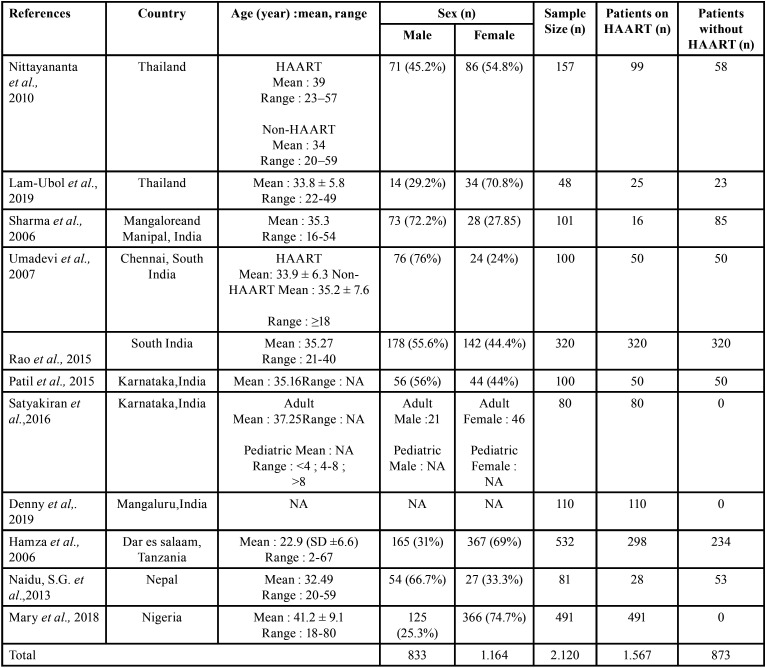
Sample size in each included studies based on geographical variation.

This review discovered 11 articles, 10 of them were cross sectional studies and only one was cohort studies. Most populations were from Asia (India; n = 811, Thailand; n =205, and Nepal; n = 81), while the remainder were from Africa, Tanzania (n = 532) and Nigeria (n = 491) ( Table 1, Table 2).

**Table 2:**
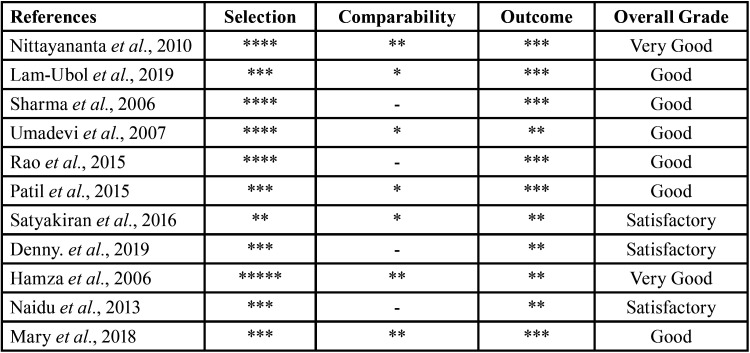
Newcastle-Ottawa Scale for Assessing Quality of Cross-sectional Study.

-Overall prevalence

**Figure 2:**
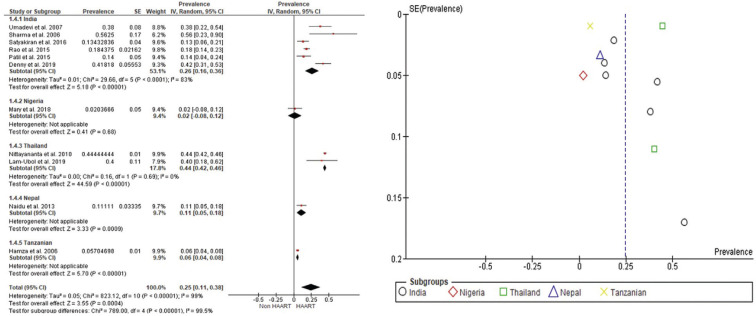
Forest plot and funnel plot of oral pigmentation prevalence in HIV-seropositive patients undergoing HAART.

The meta-analysis evaluated data from eleven articles, providing eleven prevalence estimates; the estimated prevalence ranged between 6% and 56% (Fig. 2). Forest plot analysis showed the overall pooled prevalence estimates of oral hyperpigmentation in HIV patients taking HAART was 25% (95% CI: 11%, 38%) with a high level of heterogeneity (I2= 99%).

-Subgroup Analysis

From all selected studies, the prevalence of oral hyperpigmentation was found to be distributed in certain geographic locations (Fig. 2), including India (n=6), Nigeria (n=1), Thailand (n=2), Nepal (n=1), and Tanzania (n=1). The pooling of all studies from various geographic areas presented an estimate of 25% (95% CI: 11, 38; I2=99%). The estimated prevalence of oral hyperpigmentation in India ranged between 14% and 56%. Meta-analysis displayed a random-effects pooled prevalence of 26% (95% CI: 16%, 36%) in India, with a high level of heterogeneity (I2 =83%). Meanwhile in Thailand, the meta-analysis consecutively showed the random-effects pooled prevalences was 44% (95% CI: 42%, 46%) with low heterogeneity (I2 =0%). Since there is only one study in Nigeria, Nepal, and Tanzania, meta-analysis was not applicable (2,3,6,10,19-24).

-Risk of Bias

A result of risk of bias of the included articles is provided in Table 2, along with details of the ratings for each study. The majority of the included studies had a moderate to low risk of bias, according to the article quality. Eight studies (73%) were considered to be at low risk of bias for both selection and comparability measurement, while three studies (27%) were considered to be at moderate risk of bias for selection and comparability measurement (16).

-Clinical Feature of Oral Hyperpigmentation

Based on the articles that have been analyzed, the clinical features of oral hyperpigmentation were observed on the buccal, tongue, and attached gingiva especially in pediatric patient (10,22).

-Antiretroviral Regimen Used in the Studies

Eight studies reported the type and duration of antiretroviral drugs used in the study sample, three of which did not report relevant information. Details of this data can be seen in Table 3. The most widely used type of antiretroviral drug was from the NRTI group (n=7). The shortest reported duration of using HAART was for < 4 months by Hamza *et al*. (2006), while the longest duration of HAART use was reported by Satyakiran *et al*. (2016) which was more than 8 years (10,21)

**Table 3:**
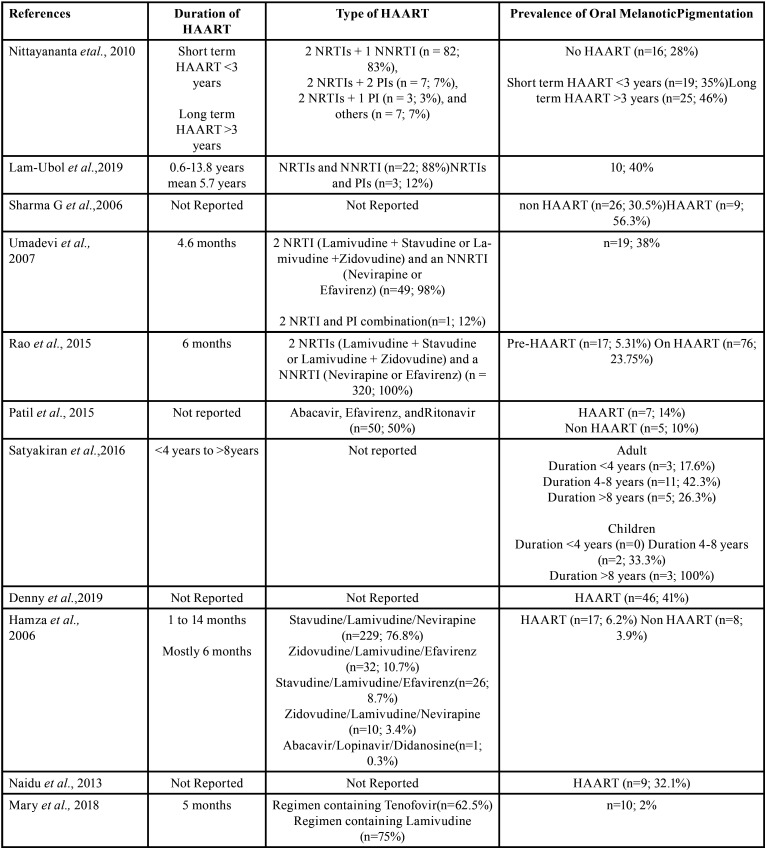
Duration and Type of Highly Active Antiretroviral Therapy Used in the Studies.

## Discussion

Oral hyperpigmentation is the deposition process of pigment in tissues pertaining to the mouth (25). The process of oral hyperpigmentation can be caused by various things either physiologic or pathologic, where pathologic condition may be resulted either from endogenous or exogenous factors (25). Ten to twenty percent of oral hyperpigmentation is reported to be the result of drug consumption including antiretroviral treatment. Although it will subside after the discontinuation of the causative medication, it becomes a great concern in patients with chronic diseases who require polypharmacy and long-term therapy such as in HAART (12,26).

In this study, the current meta-analysis reveals that the prevalence of oral hyperpigmentation among HIV patients under HAART is 25% (95% CI: 11%, 38%). This study indicated that oral hyperpigmentation can be observed in patients under HAART (e.g. 25 of 100 patients) which require anticipation to maintain the adherence to the antiviral drug regimen. This is the first pooled prevalence for oral hyperpigmentation in HAART patients presenting the highest random-effects pooled prevalence was in Thailand at 44% (95% CI: 42%, 46%) and followed by India at 24%. However, no pooled prevalence was analyzed in the populations in Nepal (11%), Tanzania (6%), and Nigeria (2%) since there is only one study in each of the three countries. The result in this pooling prevalence is varied may be due to the type and duration of HAART used in study population (27,28).

The most widely used type of ARV was from the NRTI group (n=7), which can be seen from the use of Tenofovir, Lamivudine, Abacavir, Stavudine, Zidovudine, and Didanosine. Initially, HAART therapy commonly combined NRTI and NNRTI agents to inhibit the activity of reverse transcriptase (RT) in HIV viruses as a sole target. Afterwards, more HAART are the combination of NNRTI/NRTI with protease inhibitors-aiming at interfering both HIV virus proliferations and RT activities (26). However, the use of various drugs from the NRTI group, such as Zidovudine, Lamivudine, Abacavir, Tenofovir, and Emtricitabine has been reported by various studies to have side effects related to oral hyperpigmentation in HIV patients. Zidovudine has been linked to hyperpigmentation in the nails, skin, and mouth (6,27). Mallagray-Montero (2022) in his study also found that the combined treatment of abacavir sulfate/lamivudine/zidovudine had an effect in causing oral pigmentation (28).

**Figure 3:**
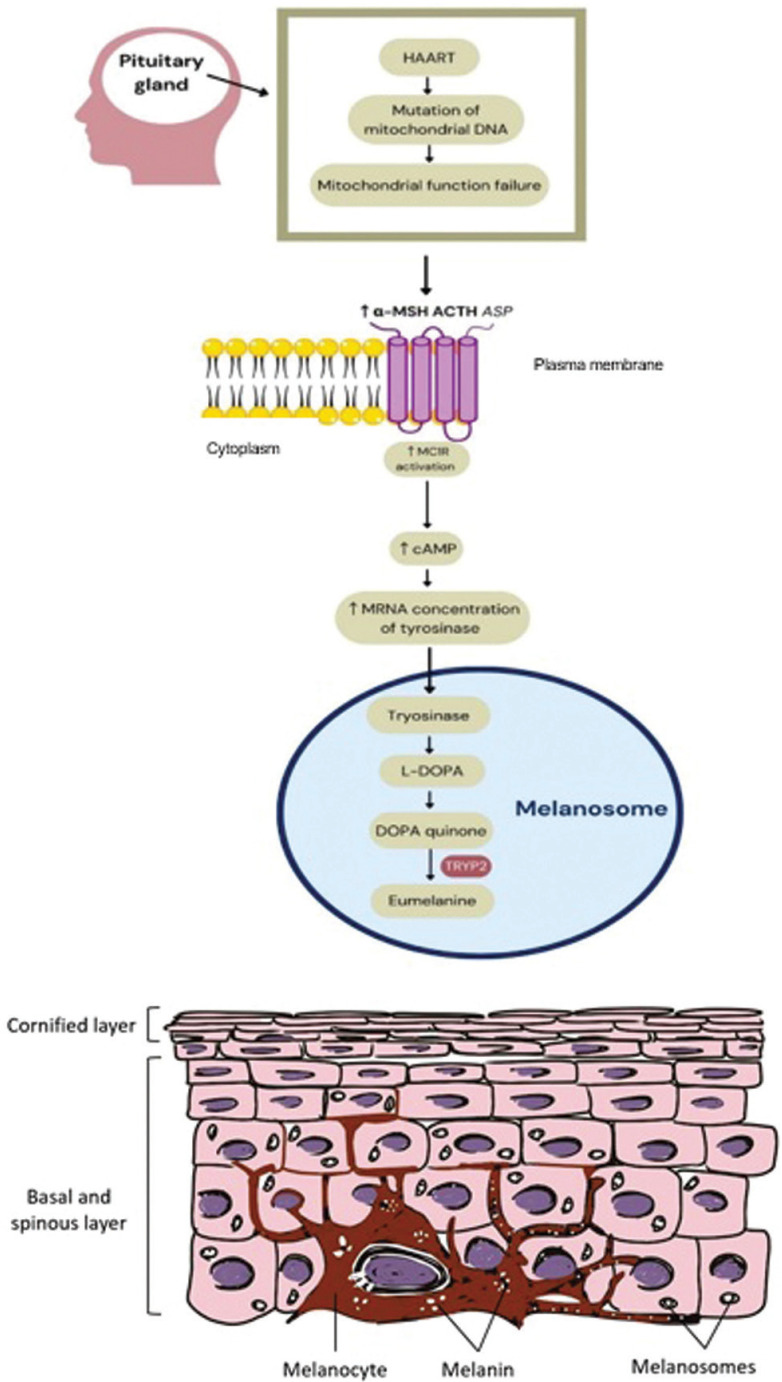
Melanin formation and transfer of melanin pigment to keratinocytes (32).

The mechanism of HAART in the increased production of melanin is illustrared in Figure 3. HAART in the NRTI group works by being incorporated into viral DNA to cause chain termination via polymerase gamma (Pol-γ). In neighboring cells, high processivity of Pol-γ and ART exposure lead to mutation of mitochondrial DNA (mtDNA) thus inducing mitochondrial function failure and increased oxidative stress (29). Inside melanosome, increased reactive oxygen species may induce polymerization of dopachrome decarboxylation product (reactive quinones) which finally leads to the formation of the brown/black eumelanin (30). As melanocyte stimulating drugs, the mechanism of action of these antiretrovirals is also postulated from an increase in melanin synthesis as an outcome of increased melanocyte-stimulating hormone secretion (a-MSH) though the process has yet to be elucidated. High oxidative stress and increased melanocyte-stimulating hormone secretion (a-MSH) would elevate the activation of melanocortin 1 receptor (MC1R) on membrane cell of melanocyte resulting in the increased mRNA concentration of tyrosinase and produced melanin (28,31). Other group of HAARTs such as NNRTI and PI is also reported with mitochondrial dysfunction and cellular stress that may also lead to increased melanin production (29).

Although oral hyperpigmentation may appear immediately after drug administration or over a longer period such as days or even years, the duration of ART has an impact on the prevalence of oral hyperpigmentation regardless of the type of antiretroviral drug used (28). It is supported by Nittayananta (2010), who found that HIV-infected subjects who took ART for a longer period of time than those who took it for a shorter period of time had a higher risk of developing oral hyperpigmentation (6). Of all 11 studies, only 7 studies reported the duration of HAART among the HIV patients where the study with the shortest duration showed the lowest oral hyperpigmentation prevalence (2,10,19,21,22). However, no study has been conducted to investigate the correlation between HAART type, duration and the degree of oral hyperpigmentation.

**Figure 4:**
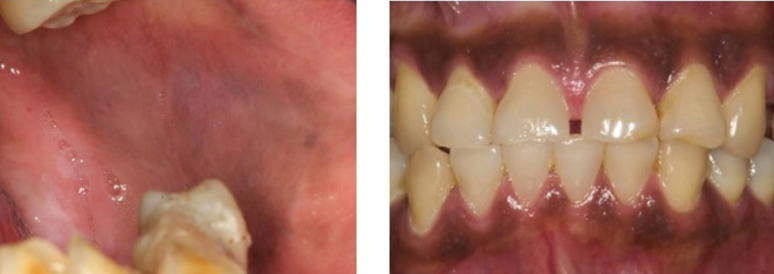
Diffuse light (left) and homogenous (right) pigmentation (34).

Clinically, oral hyperpigmentation lesions are asymptomatic and can appear in various sizes and shapes (Fig. 4). It manifests as light to dark brown macules, single or multiple, well or ill-defined, with smooth surfaces (12). It can affect any part of the oral mucosa, such as the gingiva, palate, labial mucosa, tongue, alveolar ridge, floor of the mouth but most commonly the buccal mucosa (27,33). It may affect the patient’s quality of life, especially self-confidence, where hyperpigmentation interferes with the patient’s aesthetics based on location of lesion in the oral cavity (12). Hyperpigmentation in HAART patients may either appear concurrently in skin, nails oral mucosa or solely presents in the oral cavity. Only two included studies reported the characteristics of oral hyperpigmentation that are Satyakiran *et al*.’s (2016) mentioning oral hyperpigmentation among HIV patients under HAART was found in the buccal, tongue, and attached gingiva especially in pediatric patient, and Mary *et al*. (2018) mentioning the lesion on the tongue and buccal mucosa (10,22).

HAART induced pigmentation in the oral cavity may be caused by the diffusion of drug metabolite from the capillary in the lamina propria to the basal layer where melanocytes are present. Sole presentation in the oral cavity may be determined by several factors including the number and melanogenic activity of the melanocytes in the basal cell layer of the epithelium, differences in number, size, and distribution of melanosomes, differences in the type of melanins, and the masking effect of heavily keratinized epithelium (35,36). Pigmentation due to drugs, antiretroviral drugs investigated in this review particularly, is known to be reversible and relatively dose-dependent where melanin disappears upon keratinocyte terminal differentiation in epithelial layer. The accumulation of melanin will remain as the causative drugs continued (37-39).

Potential influences on prevalence estimates were investigated using subgroup analyses. Where studies allowed, we descriptively compared prevalence estimates by location within studies. We then assessed the influence on estimates of the following study-level variables identified a priori as potential sources of variation in the estimates of prevalence: 1) risk of bias, and 2) geographical location. We undertook an initial descriptive analysis of the studies. Heterogeneity between estimates was assessed using the I2 statistic, which describes the percentage of variation not because of sampling error across studies. An I2 value above 75% is categorized as high as observed in India (83%) indicating that the source of heterogeneity was not geographical location. The high level of heterogeneity in pooled prevalence estimates may have been due to varied study populations such as age and gender or type and duration of HAART. However, further analysis could not be performed due to unreported prevalence based on age, gender, type of HAART, and duration of HAART. Based on Figure 2, it can be observed that funnel plots appear in asymmetry. This may result from smaller studies which presented larger prevalence than the number of populations. True heterogeneity may also lead to the asymmetry (32,40). One of the strengths of this study is that we reported a detailed characterization of oral hyperpigmentation and used a systematic meta-analysis to analyze the prevalence of oral hyperpigmentation caused by HAART using a systematic meta-analysis. However, there are limitations that must be addressed in the result of this review. Although being examined by three independent reviewers, the result of this review may be biased due to the quality assessment of the individual studies. Out of a total of 11 studies, 3 of them had a moderate risk of bias and many of them were due to low scores in selection and comparability domains of quality scales. Moreover, because the articles included were predominantly cross-sectional studies and there was only 1 cohort study, it is not possible to point out the main factors related to a higher prevalence of oral hyperpigmentation in some regions.

## Conclusions

In summary, in this systematic review and meta-analysis, we confirmed that concurrent oral hyperpigmentation was present in about one-fifth of the studies population. This review is a preliminary study showing an increased prevalence of oral hyperpigmentation with the use of HAART in HIV patients. Further studies are required to investigate the correlation between the duration and type of HAART with pigmentation. In addition, a large number of standardized studies are also needed to reduce heterogeneity.

## References

[1] Rumbwere Dube BN, Marshall TP, Ryan RP, Omonijo M (2018). Predictors of human immunodeficiency virus (HIV) infection in primary care among adults living in developed countries: A systematic review. Syst Rev.

[2] Rao KVSE, Chitturi RT, Kattappagari KK, Kantheti LPC, Poosarla C, Baddam VRR (2015). Impact of highly active antiretroviral therapy on oral manifestations of patients with human immunodeficiency virus/acquired immuno deficiency syndrome in South India. Indian J Sex Transm Dis.

[3] Patil N, Chaurasia VR, Babaji P, Ramesh D, Jhamb K (2015). SAM. The effect of highly active antiretroviral therapy on the prevalence of oral manifestation in human immunodeficiency virus- infected patients in Karnataka, India. Eur J Dent.

[4] Parekh BS, Ou CY, Fonjungo PN, Kalou MB, Rottinghaus E, Puren A (2018). Diagnosis of Human Immunodeficiency Virus Infection. Clin Microbiol Rev.

[5] Feria MG, Taborda NA, Hernandez JC, Rugeles MT (2018). HIV replication is associated to inflammasomes activation, IL-1β, IL-18 and caspase-1 expression in GALT and peripheral blood. PLoS One.

[6] Nittayananta W, Talungchit S, Jaruratanasirikul S, Silpapojakul K, Chayakul P, Nilmanat APN (2010). Effects of long-term use of HAART on oral health status of HIV- infected subjects. J Oral Pathol Med.

[7] Reeves DB, Duke ER, Wagner TA, Palmer SE, Spivak AM, Schiffer JT (2018). A majority of HIV persistence during antiretroviral therapy is due to infected cell proliferation. Nat Commun.

[8] Vella S, Schwartländer B, Sow SP, Eholie SP, Murphy RL (2012). The history of antiretroviral therapy and of its implementation in resource-limited areas of the world. Aids.

[9] Ponnam SR, Srivastava G, Theruru K (2012). Oral manifestations of human immunodeficiency virus in children: An institutional study at highly active antiretroviral therapy centre in India. J Oral Maxillofac Pathol.

[10] Satyakiran G, Bavle R, Alexander G, Rao S, Venugopal R, Hosthor S (2016). A relationship between CD4 count and oral manifestations of human immunodeficiency virus-infected patients on highly active antiretroviral therapy in urban population. J Oral Maxillofac Pathol.

[11] Abe OE, Fagbule OF, Olaniyi OO, Adisa AO, Gbolahan OO (2021). Orofacial lesions associated with long-term highly active antiretroviral therapy among hiv-seropositive adults in ibadan, nigeria. Pan Afr Med J.

[12] Binmadi NO, Bawazir M, Alhindi N, Mawardi H, Mansour G, Alhamed S (2020). Medication- induced oral hyperpigmentation: A systematic review. Patient Prefer Adherence.

[13] Abduljabbar T, Vohra F, Akram Z, Ghani SMA, Al-Hamoudi N, Javed F (2017). Efficacy of surgical laser therapy in the management of oral pigmented lesions: A systematic review. J Photochem Photobiol B Biol.

[14] Page MJ, McKenzie JE, Bossuyt PM, Boutron I, Hoffmann TC, Mulrow CD (2021). The PRISMA 2020 statement: An updated guideline for reporting systematic reviews. BMJ.

[15] Modesti PA, Reboldi G, Cappuccio FP, Agyemang C, Remuzzi G, Rapi S (2016). Panethnic differences in blood pressure in Europe: A systematic review and meta-analysis. PLoS One.

[16] Herzog R, Álvarez-Pasquin MJ, Díaz C, Del Barrio JL, Estrada JM, Gil Á (2013). Are healthcare workers intentions to vaccinate related to their knowledge, beliefs and attitudes? A systematic review. BMC Public Health.

[17] Anchala R, Kannuri NK, Pant H, Khan H, Franco OH, Di Angelantonio E (2014). Hypertension in India: A systematic review and meta-analysis of prevalence, awareness, and control of hypertension. J Hypertens.

[18] Alosh M, Huque MF, Koch GG (2015). Statistical Perspectives on Subgroup Analysis: Testing for Heterogeneity and Evaluating Error Rate for the Complementary Subgroup. J Biopharm Stat.

[19] Umadevi KMR, Ranganathan K, Pavithra S, Hemalatha R, Saraswathi TR, Kumarasamy N (2007). Oral lesions among persons with HIV disease with and without highly active antiretroviral therapy in southern India. J Oral Pathol Med.

[20] Lam-ubol A, Rungsiyanont S, Vacharotayangul P, Sappayatosok K, Chankanka O (2019). Oral manifestations, salivary flow rates and Candida species in Thai HIV-infected patients. J Clin Exp Dent.

[21] Hamza OJM, Matee MIN, Simon ENM, Kikwilu E, Moshi MJ, Mugusi F (2006). Oral manifestations of HIV infection in children and adults receiving highly active anti-retroviral therapy [HAART] in Dar es Salaam, Tanzania. BMC Oral Health.

[22] Mary EO, Abiola OA, Titilola G, Mojirayo OO, Sulaimon AA (2018). Prevalence of HIV related oral lesions in people living with HIV and on combined antiretroviral therapy: A Nigerian experience. Pan Afr Med J.

[23] Denny CE, Ramapuram J, Bastian TS, Binnal A, Natarajan S, Sujir N (2019). Oral and systemic comorbidities and its relation to cluster of differentiation 4 counts in human immunodeficiency virus patients on highly active antiretroviral therapy: An observational study. World J Dent.

[24] Naidu SG, Thakur R, Singh AK, Rajbhandary S, Mishra RK, Sagtani A (2013). Oral lesions and immune status of HIV infected adults from eastern Nepal. J Clin Exp Dent.

[25] Sreeja C, Ramakrishnan K, Vijayalakshmi D, Devi M, Aesha I, Vijayabanu B (2015). Oral pigmentation: A review. J Pharm Bioallied Sci.

[26] Lu DY, Wu HY, Yarla NS, Xu B, Ding J, Lu TR (2017). HAART in HIV/AIDS Treatments: Future Trends. Infect Disord - Drug Targets.

[27] Shoubin M, Kandasamy M, Gopalan K, Vellaisamy SG, Manickam N (2018). Zidovudine-induced pigmentation on skin, nail and oral cavity - A study on 119 patients. J Pakistan Assoc Dermatologists.

[28] Mallagray-Montero MDC, Moreno-López LA, Cerero-Lapiedra R, Castro-Janeiro M, Madrigal- Martínez-pereda C (2022). Medication related to pigmentation of oral mucosa. Med Oral Patol Oral y Cir Bucal.

[29] Schank M, Zhao J, Moorman JP, Yao ZQ (2021). The impact of hiv-and art-induced mitochondrial dysfunction in cellular senescence and aging. Cells.

[30] Denat L, Kadekaro AL, Marrot L, Leachman S (2014). A-MZA. Melanocytes as Instigators and Victims of Oxidative Stress. J Invest Dermatol.

[31] Swope VB, Abdel-Malek ZA (2016). Significance of the melanocortin 1 and endothelin B receptors in melanocyte homeostasis and prevention of sun-induced genotoxicity. Front Genet.

[32] Doleman B, Freeman SC, Lund JN, Williams JP, Sutton AJ (2020). Funnel plots may show asymmetry in the absence of publication bias with continuous outcomes dependent on baseline risk: presentation of a new publication bias test. Res Synth Methods.

[33] Chandran R, Feller L, Lemmer J, Khammissa RAG (2016). HIV-Associated Oral Mucosal Melanin Hyperpigmentation: A Clinical Study in a South African Population Sample. AIDS Res Treat.

[34] Rosebush MS, Briody AN, Cordell KG (2019). Black and Brown: Non-neoplastic Pigmentation of the Oral Mucosa. Head Neck Pathol.

[35] Feller L, Masilana A, Khammissa RAG, Altini M, Jadwat Y, Lemmer J (2014). Melanin: The biophysiology of oral melanocytes and physiological oral pigmentation. Head Face Med.

[36] Lock JY, Carlson TL, Wang CM, Chen A, Carrier RL (2018). Acute Exposure to Commonly Ingested Emulsifiers Alters Intestinal Mucus Structure and Transport Properties. Sci Rep.

[37] Singh S, Rai T (2014). A case of zidovudine induced pigmentation on palms and soles. Indian Dermatol Online J.

[38] Moreiras H, Seabra MC, Barral DC (2021). Melanin transfer in the epidermis: The pursuit of skin pigmentation control mechanisms. Int J Mol Sci.

[39] Giménez García RM, Molina SC (2019). Drug-induced hyperpigmentation: Review and case series. J Am Board Fam Med.

[40] Von Hippel PT (2015). The heterogeneity statistic I2 can be biased in small meta-analyses. BMC Med Res Methodol.

